# Cell permeable HMGB1-binding heptamer peptide ameliorates neurovascular complications associated with thrombolytic therapy in rats with transient ischemic stroke

**DOI:** 10.1186/s12974-018-1267-5

**Published:** 2018-08-23

**Authors:** Miaodan Li, Shumin Chen, Xue Shi, Chenfei Lyu, Yongfang Zhang, Miaoqin Tan, Chen Wang, Nailiang Zang, Xiaoxi Liu, Yafang Hu, Jiangang Shen, Liang Zhou, Yong Gu

**Affiliations:** 1 Department of Neurology, Nanfang Hospital, Southern Medical University, Guangzhou, Guangdong 510515 People’s Republic of China; 2 School of Chinese Medicine, The University of Hong Kong, Hong Kong, People’s Republic of China

**Keywords:** Ischemic stroke, Tissue-type plasminogen activator, Blood–brain barrier, Hemorrhagic transformation, HMGB1, HMGB1-binding heptamer peptide, Inflammation

## Abstract

**Background:**

Blood–brain barrier (BBB) breakdown and inflammatory responses are the major causes of tissue-type plasminogen activator (tPA)-induced hemorrhagic transformation (HT), while high-mobility group box 1 (HMGB1) exacerbates inflammatory damage to BBB during the process of brain ischemia/reperfusion. This study aimed to investigate the change of HMGB1 after thrombolytic therapy and whether blocking HMGB1 could ameliorate the neurovasculature complications secondary to tPA treatment in stroke rats.

**Methods:**

Sera from acute stroke patients and rats with thrombolytic therapy were collected to investigate HMGB1 secretion. Male Sprague-Dawley rats with 2 h or 4.5 h middle cerebral artery occlusion were continuously infused with tPA followed by administration of membrane permeable HMGB1-binding heptamer peptide (HBHP). The mortality rate, neurological score, HT, brain swelling, BBB permeability, and inflammatory factors were determined.

**Results:**

The results revealed that HMGB1 levels were elevated in both stroke patients and rats after tPA treatment. Blocking HMGB1 signaling by HBHP in the rat model of 4.5 h brain ischemia significantly attenuated tPA-related complications, including mortality rate, the degree of hemorrhage, brain swelling, neurological deficits, BBB impairment, microglia activation, and the expressions of inflammatory cytokines.

**Conclusions:**

tPA treatment might induce HMGB1 secretion while blocking HMGB1 with HBHP could markedly reduce the risk of thrombolysis-associated brain hemorrhage and mortality through attenuating BBB damage and inflammatory reactions. These results indicate that HMGB1 may potentiate the risk of HT in tPA administration and that blocking HMGB1 signaling would be helpful in preventing complications brought by thrombolysis in ischemic stroke.

**Trial registration:**

http://www.chictr.org.cn. Unique identifier: ChiCTR-OOC-16010052. Registered 30 November 2016.

**Electronic supplementary material:**

The online version of this article (10.1186/s12974-018-1267-5) contains supplementary material, which is available to authorized users.

## Background

Ischemic stroke is the second leading cause of death worldwide while the therapeutic approaches are limited [1]. Tissue-type plasminogen activator (tPA) is the only FDA-approved drug for ischemic stroke, but its use is finite due to the narrow therapeutic time window (within 3 h or 4.5 h) and the increased risk of severe neurovascular complications, such as hemorrhagic transformation (HT) and edema [2], resulting in the fact that more than 95% of stroke patients cannot benefit from thrombolytic therapy [3]. The leakage of blood–brain barrier (BBB) is a critical factor causing tPA-associated HT and edema [4]. Therefore, exploring a combination therapy that can preserve BBB integrity is a promising therapeutic strategy to reduce the risk of neurovasculature complications.

Although the mechanisms underlying BBB breakdown and tPA-induced neurovascular complications are not fully understood, it has been suggested that they occur as a result of exaggeration of neuroinflammation [5]. Danger signals, such as high-mobility group box 1 (HMGB1) and ATP, were released into the environment from dying cells, leading to the activation of microglia and the expression of inflammatory factors in ischemic brain [6]. HMGB1, a non-histone DNA-binding protein localized in the nucleus, can be either actively secreted by immune cells or passively released from necrotic cells in response to infections and tissue injuries [7, 8]. Studies conducted by us and others revealed that the binding of extracellular HMGB1 to pattern recognition receptors of microglia could induce a significant elevation of cytokines expression, thereby eliciting inflammatory responses during brain ischemia/reperfusion [9, 10]. Moreover, HMGB1 serves as an extracellular inflammatory cytokine and contributes to neuronal injury and BBB disruption. HMGB1 level was elevated in patients with ischemic stroke [11, 12]. Anti-HMGB1 monoclonal antibody reduces infarct volume by 90% and potently reduces BBB permeability in stroke animal models [13, 14]. Administration of low-dose HMGB1-binding heptamer peptide (HBHP) significantly suppressed HMGB1-mediated neuronal damage [15]. But the relationship between HMGB1 and tPA-induced complications is unclear. Given that neuroinflammation contributes to the BBB disruption and tPA’s neurovascular complications, we logically hypothesize that HMGB1 may potentiate the risk of tPA-associated complications, while blocking HMGB1-induced inflammation could protect BBB integrity, subsequently reducing tPA-induced hemorrhage during thrombolytic therapy for ischemic stroke.

## Methods

### Patients

Participants were selected from acute stroke patients admitted to the Department of Neurology, Nanfang Hospital, Southern Medical University. Enrollment criteria included subjects who were diagnosed with acute cerebral ischemia caused by occlusion of middle cerebral artery and met the criteria for thrombolysis. Excluded patients were those suffering from acute inflammatory illness, autoimmune diseases, or cancer. Patients who recently took anti-inflammatory drugs, glucocorticoid, or embolectomy were also ruled out. The study was approved by the ethics committee of the Nanfang Hospital, Southern Medical University (NO. NFEC-2016-171). Written informed consent was provided by patients or legally authorized representatives, and we obtained the consent from every participants or representatives to publish and report the data.

### Animals and ethics statement

Male Sprague-Dawley rats weighing 250~300 g were obtained from the Experimental Animal Center, Southern Medical University. All animals were housed in a standard light-dark cycle with an average temperature of 23 °C. Food and water were provided ad libitum. All procedures were approved by the committee on Animal Care and Use of Nanfang Hospital, Southern Medical University, and followed the guide and care for the use of laboratory animals published by US NIH. A total of 166 rats were employed in the study. All efforts were made to minimize the number of animals used and their pain suffered.

### Transient focal brain ischemia/reperfusion model

**Fig. 1:**
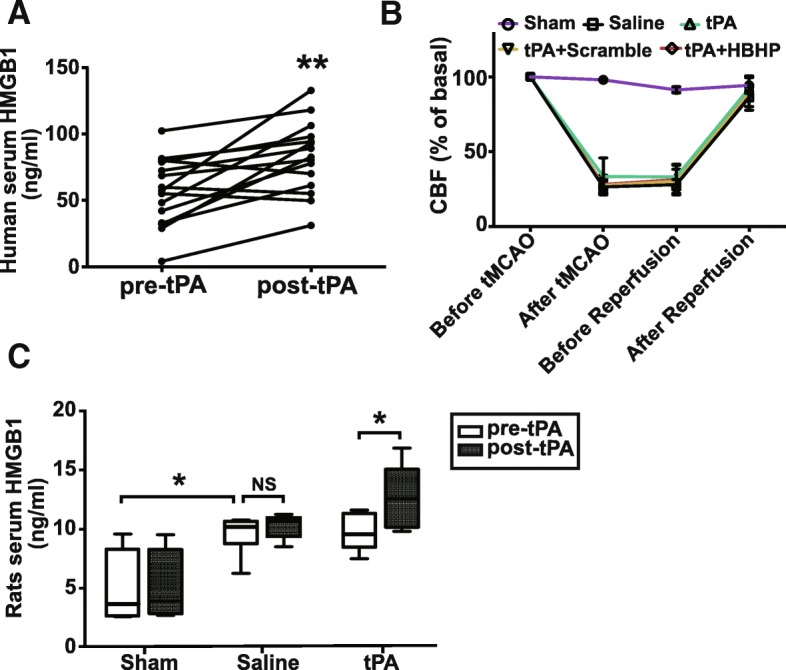
Serum HMGB1 levels were elevated after thrombolysis in stroke patients and rats. **a** ELISA data of HMGB1 concentrations in patients’ sera collected pre- and 2 h post-thrombolysis (*n* = 15). **b** Cerebral blood flow (CBF) of each rat subjected to ischemia/reperfusion was detected by laser-Doppler and expressed as percentage of basal level. ***c****. ELISA* result of serum HMGB1 levels at 30 min pre- and 4 h post-treatment of vehicle saline or tPA in 4.5 h MCAO rats (*n* = 6). NS: not significant; *P < 0.05, **P < 0.01. Data were shown as mean ± SD

Middle cerebral artery occlusion (MCAO) model was employed as we previously described [16] to induce transient focal brain ischemia/reperfusion. Briefly, rats were anesthetized by inhalation of 5% isoflurane and maintained by 2% isoflurane in a mixture of 70% N2O and 30% O2. A piece of 3/0 monofilament nylon suture with its tip lapped round by silicone was introduced via lumen of right external carotid artery stump to embed into right anterior cerebral artery so that right MCA was occluded at its origin. Rats in sham-operated group underwent the same procedures except occluding the MCA. The temperature was maintained at 37 ± 0.1 °C until animals woke up completely. Animal operations and preclinical stroke study were followed ARRIVE guidelines [17]. Cerebral blood flow velocity of the right MCA territory (core cortex, 2 mm posterior and 6 mm lateral to the bregma) was assessed by the laser Doppler blood flow assessment (Moor Instruments, Wilmington, DE). After 2 h or 4.5 h occlusion, suture was removed to induce blood reperfusion whose flow velocity was monitored again (Fig. 1b). To confirm the success of MCAO, a 2-mm-thick brain coronal section 6 mm away from the tip of the frontal lobe was stained with 2,3,5-triphenyltetrazolium chloride (TTC) [18].

### Detection of HMGB1 and interleukin (IL)-1β in sera from patients and rats

Blood samples from patients were collected pre- and 2 h post-tPA treatment for self-comparison. Rat blood samples were collected pre- and 4 h post-tPA administration. Sera from patients and rats were obtained and the levels of HMGB1 were detected by enzyme-linked immunosorbent assay (ELISA) kits (Chondrex, Redmond, WA). Meanwhile, sera were incubated with Protein A/G MagBeads (GenScript, Piscataway, NJ) to remove immunoglobins followed by Western blot to observe HMGB1 levels. IL-1β in the sera from rats at 8 h after ischemia were also detected by ELISA kits (eBioscience, Austria). Tests were performed by investigators blinded to the origin of samples.

### Experimental groups and drug treatment

MCAO rats were randomly divided into four groups with randomized number table and, respectively, administered saline, tPA only, tPA plus scramble peptide or tPA plus HBHP. tPA (Actilyse, 5 mg/kg, Boehringer Ingelheim, German) was injected (10% bonus, 90% continuous infusion for 30 min) via right femoral vein of MCAO rats. The suture was withdrawn to induce reperfusion at 15 min after tPA injection. Different doses of TAT-HBHP (YGRKKRRQRRR-HMSKPVQ, 1, 5, 10 mg/kg) were intravenously administered after tPA treatment within 30 min (Fig. 3a) [15, 19]. TAT-scramble peptide (YGRKKRRQRRR-PMQSKHV, 5 mg/kg) was employed as vehicle control. Peptides were synthesized by ChinaPeptides, Shanghai, China.

### Measurement of survival rate and neurological deficit

Rats were monitored by video camera after surgery, and the time of death was recorded. Neurological deficits at 24 h after MCAO were assessed using modified Neurological Severity Score (mNSS) [20]. Neurological function was graded on a series of scales from 0 to 18 with higher scores indicating more severe neurologic deficits. Tests were independently performed by two investigators blinded to animal grouping.

### Measurement of brain swelling and brain hemorrhage

At 24 h after ischemia, rats were transcardially perfused with cold PBS. The hemispheric area of each 2-mm-thick brain slice was measured to calculate the brain swelling by using ImageJ software (NIH, Bethesda, MD). The swelling index was calculated using the following equation: extent of brain swelling = (volume of ischemia ipsilateral hemisphere/volume of contralateral hemisphere − 1) × 100%. After taking photographs, the brain tissues were homogenized and centrifuged. QuantiChrom Hemoglobin Assay Kit (BioAssay Systems, Hayward, CA) was used to calculate the levels of hemoglobin in supernatants at 400 nm optical density. Hemoglobin contents of hemispheres were calculated based on optical density and expressed as microgram per gram brain.

### Extravasation of Evans blue

Blood–brain barrier (BBB) integrity was assessed by measuring the extravasation of Evans blue dye. Briefly, Evans blue (2% in saline, 4 ml/kg; Sigma-Aldrich) was administered 90 min before sacrifice followed by transcardially perfused with saline to remove the residual dye from the vessels. The hemispheres were weighed and incubated in methanamide (Sigma-Aldrich) in 60 °C water bath overnight. After that, Evans blue content was determined in supernatants at 632 nm and expressed as microgram per gram brain. Gradient concentrations of Evans blue were used to build standard curve.

### Western blot analysis

Proteins were prepared from the same regions of ischemic cerebral hemisphere (bregma 0 to + 2 mm) from different treatment groups, or serum proteins after magnetic separation. Fifty micrograms of total proteins were separated on 8 to 12% SDS-PAGE gel. After blocking, the membranes were incubated overnight at 4 °C with the primary antibodies including anti-occludin (1:1000; Invitrogen, Camarillo, CA), anti-COX-2 (1:1000; CST, Beverly, MA), anti-IL-1β, anti-iNOS (1:200; Santa Cruz Biotech, CA), anti-β-actin (1:500; ZSGB-Bio, Beijing, China) or anti-HMGB1 (1:1000; Proteintech, UK), followed by incubation with horseradish peroxidase-conjugated secondary antibodies (1:5000; Santa Cruz Biotech). Bands were detected by ECL advance Western blotting detection reagents (Millipore, Billercia, MA). The band intensities were normalized to β-actin using the ImageJ.

### RNA preparation and reverse transcription-PCR

Total RNA was isolated from brains of 4.5 h MCAO rats at 8 h after ischemia onset (bregma − 1 to + 1 mm), and reverse-transcribed to complementary DNA (cDNA) using PrimeScript™ RT Master Mix Kit (Takara, Japan). cDNA samples were then amplified by quantitative real-time PCR on ABI-Prism 7500 Real-Time PCR System (Applied Biosystems, Carlsbad, CA) using SYBR® Premix Ex Taq™ II (Takara). The expression of mRNA was normalized with the internal standard β-actin.

### Histological examination

Cryosections cut from rat brains (0~2.0 mm posterior to the bregma) were immunolabeled by primary antibody against IgG (Alexa647; 1:500; Abcam, Cambridge, UK) to evaluate the permeability of BBB at 24 h. Meanwhile, sections collected at 8 h after ischemia were postfixed with 4% formaldehyde, permeabilized with Triton X-100, and blocked with donkey serum, followed by incubation with anti-Iba-1 or CD68 (a marker of activated microglia cells; 1:200; Abcam) overnight at 4 °C, then with the donkey anti-goat (1:500; Abcam) or goat anti-mouse (ZSGB-BIO) secondary antibody. Three serial sections of each brain sample were observed, nine fields were randomly selected in the brain section region surrounded by black solid line (Fig. 6d) under Olympus Fluoview laser scanning confocal microscope (Olympus, Japan). The mean counts of Iba-1- and CD68-positive cells per field under × 40 magnification were automatically calculated using Image-Pro Plus version 6.0 (Media Cybernetics, Warrendale, PA). All counts were performed blind to the investigator on coded sections.

### Statistical analysis

Online Power and Sample Size Calculators (http://powerandsamplesize.com/) was used to confirm the sample size needed for comparisons to yield statistical significance. Data were expressed as mean ± SD. Statistical significance was evaluated using one-way analysis of variance and followed by Dunnett test for two group comparisons. Nonparametric paired test was adopted for self-comparison. All statistical analyses were performed using SPSS 20.0 (IBM, Armonk, NY) or GraphPad Prism 6.05 (GraphPad, La Jolla, CA). *P* values less than 0.05 were considered to be significant.

## Results

### Treatment of tPA significantly elevates the serum HMGB1 levels in stroke patients and rats

We firstly determined whether HMGB1 secretion was changed after tPA treatment. Fifteen patients diagnosed with MCAO-induced acute ischemic stroke were enrolled in the study. The characteristics of enrolled patients were summarized in Table 1. Blood samples from patients were collected pre- and 2 h post-tPA treatment for self-comparison. ELISA data revealed that the serum HMGB1 level was significantly elevated after tPA treatment (56.31 ± 25.54 ng/mL before, vs 82.71 ± 26.72 ng/mL after, tPA treatment; *P* < 0.01; Fig. 1a).

**Table 1:** Patient characteristics

Traits		Risk factors	
Men/women	10/5	Diabetes	10
Age (mean ± SD, years)	61.93 ± 7.79	Hypertension	10
NIHSS	7.67 ± 4.05	Smoking	7
Anti-inflammatory drugs	0	Dyslipidemia	9
Glucocorticoid	0	High homocysteine	1

Then, animal studies were conducted to further clarify the change of HMGB1 release in response to thrombolysis. Although the embolic stroke model can closely mimic the clinical situation, it produces big variations of infarct size and locations among individuals [18, 21]. Transient filament occlusion model would be more stable for us to specifically investigate tPA-induced complications. A total of 166 rats were employed in the study. Only the rats that displayed more than 70% reduction of cerebral blood flow during ischemia and paralysis of the contralateral limb after reperfusion were selected (Fig. 1b). In line with the data of human study, the rat serum HMGB1 level was also found to be increased after tPA treatment (from 9.84 ± 1.62 to 12.62 ± 2.78 ng/mL; *P* < 0.05; Fig. 1c). Considering ischemic injury itself can also induce HMGB1 release, to eliminate the possibility that timely accumulated HMGB1 release after ischemia leads to HMGB1 elevation, we compared its dynamic change in stroke rats with saline injection. Results in Fig. 1c showed that HMGB1 was not significantly altered at 4 h after saline injection. Based on these evidences, it seems that tPA treatment induces HMGB1 release. To eliminate the potential interference brought by non-specificity of HMGB1 antibody in purchased ELISA kits, sera proteins were magnetic separated and immunoblotted by HMGB1 antibody. Results revealed that HMGB1 could be detected by Western blot and it was also increased after tPA treatment, although a relatively deep background was observed (Additional file 1: Figure S1).

### HBHP alleviates HT in MCAO rats with tPA treatment

**Fig. 2:**
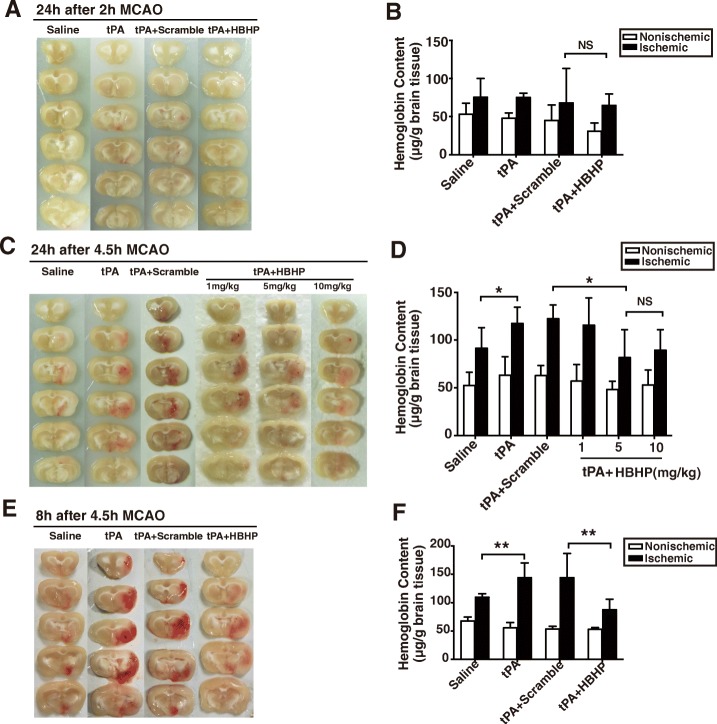
HBHP alleviated tPA-induced hemorrhage in stroke rats with 4.5 h MCAO. **a**, **b** Representative brain coronal sections showing hemorrhage and hemispheric enlargement in 2 h MCAO rats plus 22 h reperfusion with different treatments. **c**, **d** Brain slices showing hemorrhage and brain hemoglobin contents in 4.5 h MCAO rats at 24 h after ischemia onset. **e**, **f** Brain slices and hemoglobin contents in 4.5 h MCAO rats at 8 h after ischemia onset. Hemoglobin content was quantified and presented as bar graph. NS: not significant; *P < 0.05, **P < 0.01; *n* = 5–8. Data were shown as mean ± SD

To explore the correlation between elevated HMGB1 and tPA-induced complications, we next investigated whether blocking HMGB1 signaling with HBHP could improve the complication after tPA treatment. HT is the most severe side effect of thrombolytic therapy. We quantified hemorrhage volume in rats 24 h after experimental ischemia. Early tPA infusion at 2 h did not induce obvious HT (Fig. 2a, b) which is consistent with the previous finding [22]. Meanwhile, delayed tPA treatment at 4.5 h resulted in evident HT in the ischemic brain (Fig. 2c, d). Notably, co-treatment of HBHP (5 mg/kg) significantly reduced delayed tPA-induced hemorrhage, while scramble peptide and HBHP (1 mg/kg) did not. Hemoglobin quantification assay further confirmed this finding. There was no statistical difference between 5 and 10 mg/kg. Therefore, we selected 5 mg/kg as the minimum dose of maximum efficacy in the following experiments.

Since most 4.5 h-tPA rats who had more volume of brain hemorrhage might not survive to 24 h, the determination of survived rats at 24 h was inaccurate. Therefore, we quantified the contents of hemoglobin in 4.5 h-MCAO rats at 8 h after ischemia onset and found tPA administration caused severe hemorrhage, which could also be mitigated by HBHP (Fig. 2e, f). These results indicate that HBHP can alleviate tPA-induced HT in ischemic rats.

### HBHP decreases mortality of rats treated with tPA after brain ischemia

**Fig. 3:**
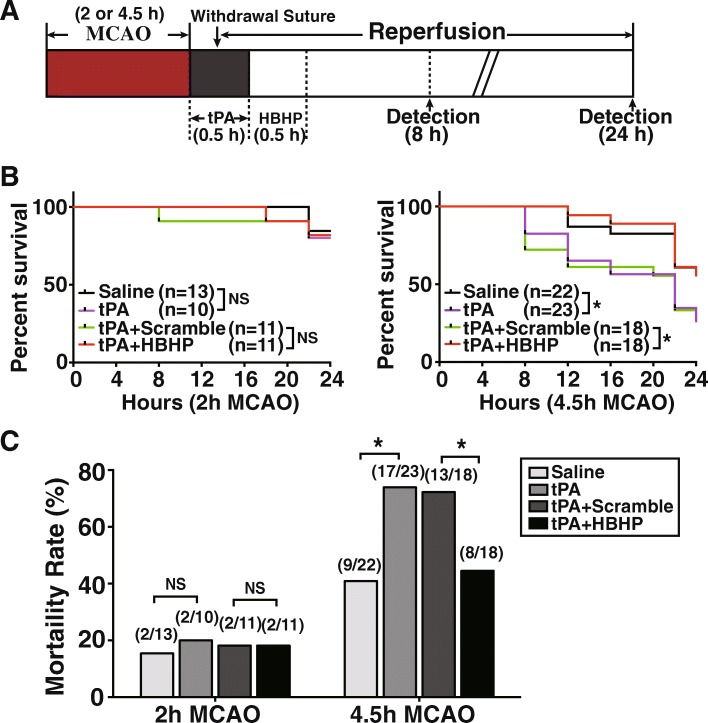
HBHP decreased the mortality of 4.5 h MCAO rats with tPA administration. **a** Schematic diagram of the overall experimental design. **b** The 24 h survival curves of rats with 2 h or 4.5 h MCAO treated with vehicle or tPA in combination of HBHP or scramble peptide. The rat number in each group was labeled. **c** The mortality of each group rats at 24 h post ischemia. Numbers in parentheses mean “the dead rats/the total number of rats” in each group. NS: not significant; *P < 0.05

Higher volume of hemorrhage in the brain brings poor prognosis. As shown in Fig. 3b, c, rats receiving 4.5 h ischemia followed by tPA administration (4.5 h-tPA) had a much higher mortality (73.91%, 17 out of 23) than the vehicle saline-treated group rats (40.91%, 9 out of 22). In contrast, HBHP markedly lowered the mortality to 44.44% at 24 h after ischemia (8 of 18; Fig. 3c) and improved 24 h survival outcome after 4.5 h ischemia followed by tPA administration (Fig. 3b). In 2 h ischemia model, the mortality was not statistically different in different treatment groups (Fig. 3c).

### HBHP improves neurological outcome and attenuates brain swelling in MCAO rats with tPA treatment

**Fig. 4:**
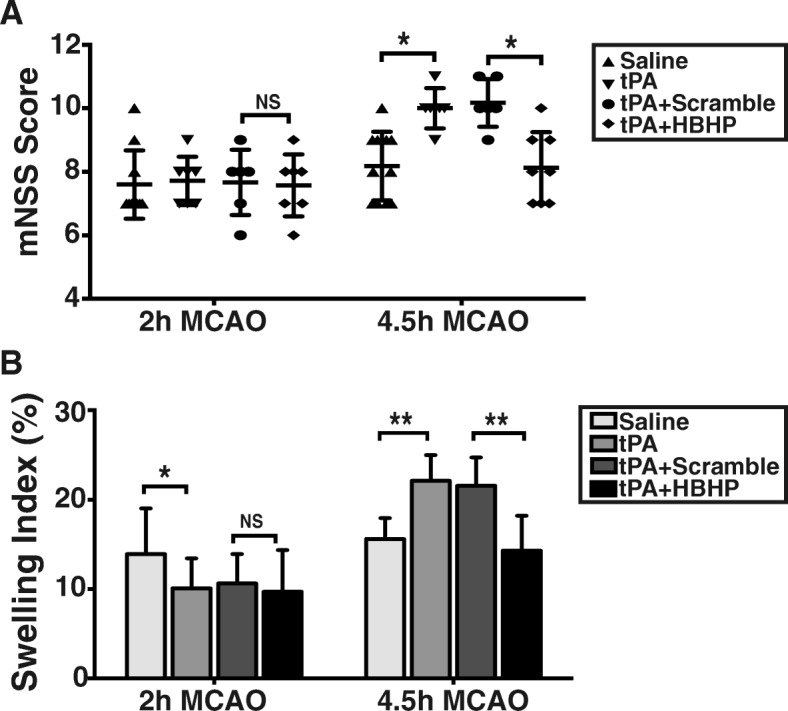
HBHP improved neurological outcome and brain swelling of 4.5 h MCAO rats with tPA treatment. **a** Modified Neurological Severity Scores (mNSS) at 24 h post ischemia. **b** The ratio of brain swelling at 24 h after ischemia of stroke rats. NS: not significant; *P < 0.05, **P < 0.01; *n* = 5–8. Data were shown as mean ± SD

Similar effects were observed for mNSS score estimation. Rats in 4.5 h-tPA group displayed more severe neurological impairment than in saline group, and the neural deficits were ameliorated by HBHP (Fig. 4a). No statistical differences in neurological outcome were noted in 2 h-MCAO groups. Expectedly, HBHP treatment significantly reduce ischemia hemispheric enlargement caused by 4.5 h-tPA (Fig. 4b). Together, these results suggest that HBHP treatment improves prognosis in rats after experimental 4.5 h ischemia followed by tPA administration.

### HBHP alleviates BBB disruption in MCAO rats with tPA treatment

**Fig. 5:**
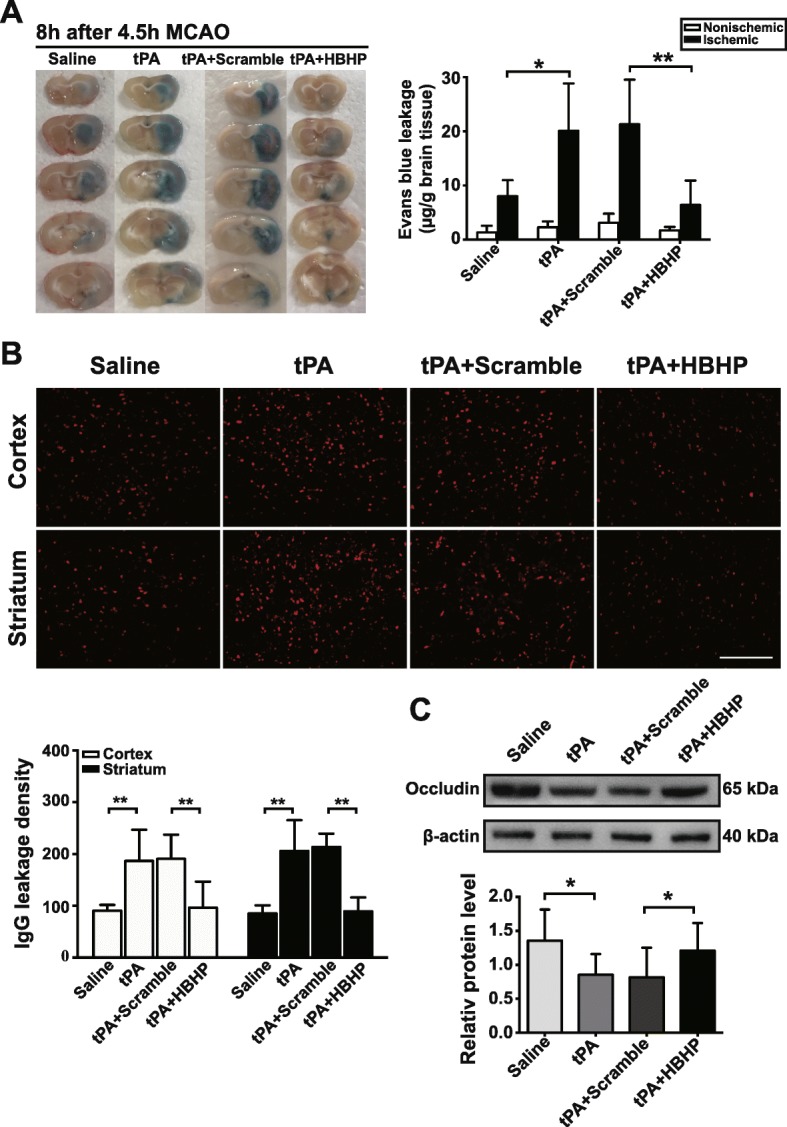
HBHP decreased the BBB permeability in tPA-treated MCAO rats. **a** Representative images of Evans blue leakage from 4.5 h MCAO rats. The contents of Evans blue leakage were measured and expressed as microgram per gram brain. *P < 0.05, **P < 0.01; *n* = 4–6. **b** Immunofluoresence staining with IgG antibody (red) in the cerebral cortex and striatum from 4.5 h MCAO rats. The leaked IgG was quantified. Scale bar = 200 μm. **P < 0.01, *n* = 3–4. **c** Western blot and quantified data of occludin in ischemic ipsilateral hemispheres from 4.5 h MCAO rats. *P < 0.05, **P < 0.01; *n* = 4. Data were shown as mean ± SD

Thrombolytic therapy has been reported to exaggerate BBB breakdown, leading to the HT in animal stroke models [4, 5]. BBB permeability in 4.5 h-MCAO rats was evaluated via the observation of the Evans blue dye and IgG leakage to brain parenchyma, as well as the loss of tight junction protein occludin. Because 2 h-MCAO did not cause high mortality and severe HT, the following experiments were conducted on 4.5 h-MCAO model. Results showed that Evans blue content in ipsilateral ischemic hemispheres was remarkably decreased in the HBHP treatment group compared with scramble group (6.409 ± 4.514 vs 21.298 ± 8.233 μg/g) (Fig. 5a). Immunofluorescence staining assay showed that a large number of IgG permeated in cortex and striatum regions of the ischemic ipsilateral hemispheres after tPA treatment, which was significantly decreased by HBHP treatment (Fig. 5b). Consistently, the tPA-induced reduction of occludin was rescued by HBHP (Fig. 5c), suggesting that HBHP protects BBB integrity in tPA-treated rats.

### HBHP downregulates inflammatory cytokine expressions and inhibits microglia activation in ischemic brains with tPA treatment

**Fig. 6:**
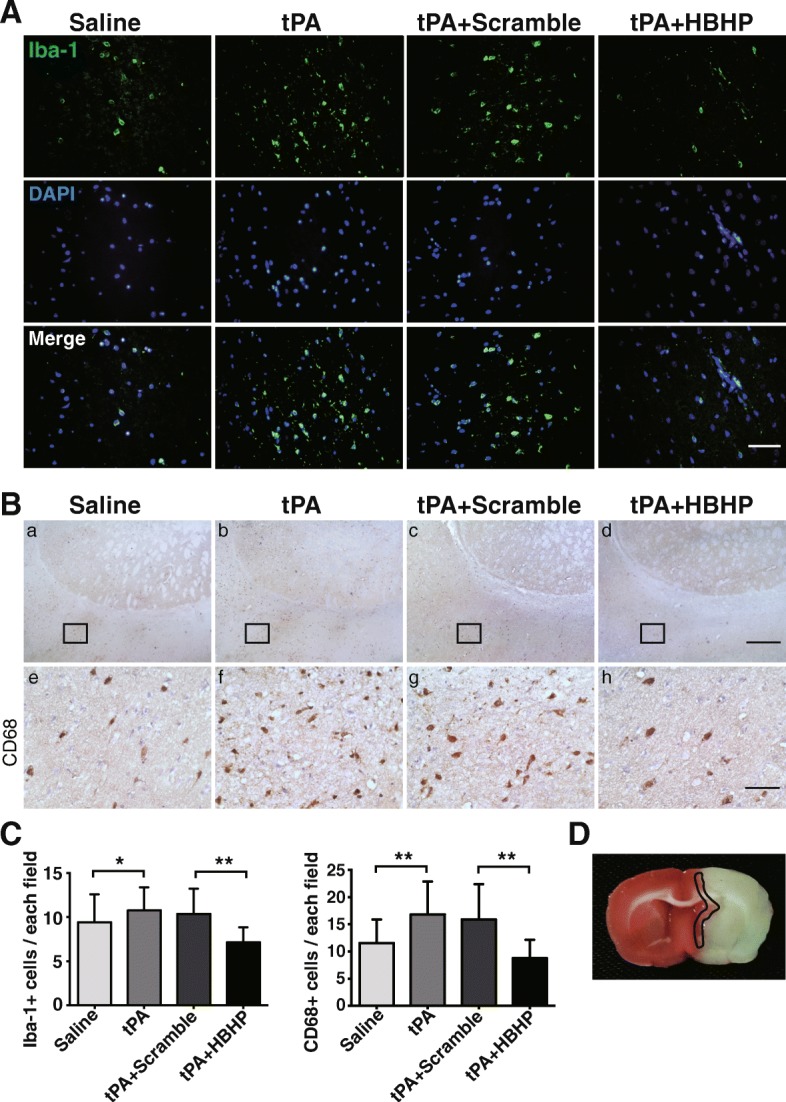
HBHP inhibited microglia activation in tPA-treated MCAO rats. **A** Representative graphs of immunofluorescence staining with anti-Iba-1 antibodies (green) and DAPI (blue) in the peri-infarct cortex from 4.5 h MCAO rats. Scale bar = 50 μm. **B** Representative graphs of immunohistochemistry staining with CD68 antibodies. The volumes of **e**–**h** were the amplification of **a**–**d**. Scale bar = 500 μm in **a**–**d** (4×); Scale bar = 50 μm in **e**–**h** (40×). **C** The number of Iba-1- and CD68-positive cells was quantified. **D** 2-3-5-Triphenyl tetrazolium chloride staining of ischemic brain indicated the position where the corresponding images were obtained. The brain region surrounded by black solid line indicates the areas where the typical Iba-1-positive cells were observed. *P < 0.05, **P < 0.01; *n* = 3–4. Data were shown as mean ± SD

**Fig. 7:**
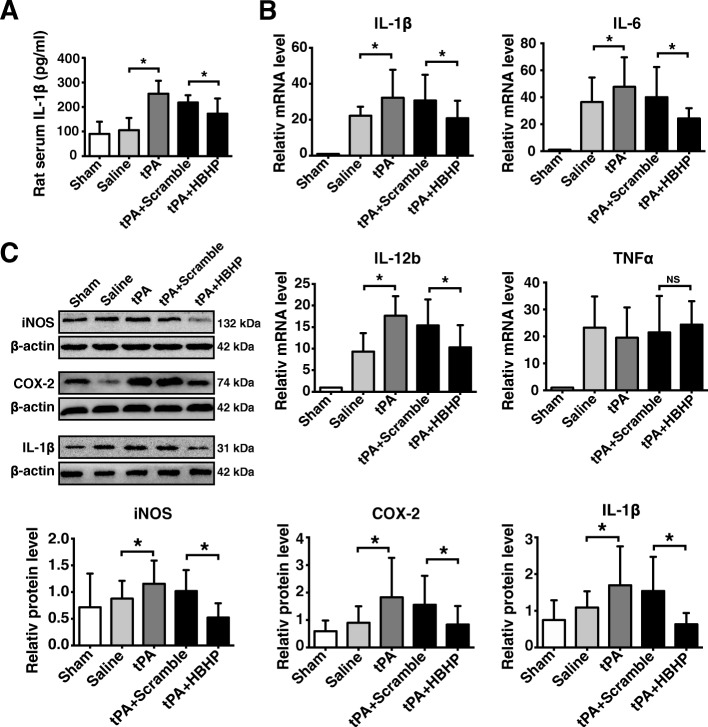
HBHP alleviated inflammatory reactions in tPA-treated stroke rats. **a** ELISA data of serum IL-1β levels in stroke rats at 8 h after ischemia, *n* = 5–7. **b** Quantitative real-time PCR analysis of mRNA expressions of IL-1β, IL-6, IL-12b, and TNF-α in the peri-infarct cortex from 4.5 h MCAO rats or in the same regions of sham-operated rats, *n* = 3–4. **c** Western blot and quantified data of iNOS, COX-2, and IL-1β in ischemic ipsilateral hemispheres at 8 h after ischemia, *n* = 3–4. NS: not significant; *P < 0.05. Data were shown as mean ± SD

Considering residential inflammation has been reported to be involved in the disruption of BBB, we finally investigated whether the protective effect of HBHP is related to inflammatory reactions. Activation of microglia and the expression of cytokines were observed at 8 h after ischemia onset. Data revealed that inflammatory cells were further activated by tPA, as evident by more CD68- and Iba-1-positive cells [23] in peri-infarct area in tPA-treated rats, which was ameliorated by HBHP treatment (Fig. 6a–c). Quantitative real-time PCR, Western blot, and ELISA assay were performed to detect cytokine expression or release. Results showed that HBHP decreased mRNA expressions of IL-1β, IL-6, and IL-12b, while TNF-α did not change after treatment (Fig. 7b). HBHP treatment significantly downregulated the protein expressions of iNOS, COX-2, and IL-1β in the ischemic hemisphere from tPA-treated rats compared with scramble control (Fig. 7c). The secretion of IL-1β, determined by ELISA, was also inhibited after HBHP treatment (Fig. 7a). Taken together, HBHP can reduce inflammatory response secondary to tPA administration, contributing to the attenuation of neurovascular complications.

## Discussion

Presently, tPA is still a highly effective therapy to salvage potentially reversible ischemic tissue and early improve neurological deficits. However, tPA application is limited by narrow therapeutic time window and severe neurovascular complications. Stroke patients with tPA treatment have about 10-fold higher risk of suffering HT than untreated patients. A recent study indicates 28% had hemorrhages in the ischemic area and 44.6% developed cerebral microbleeds using susceptibility-weighted imaging (SWI), a magnetic resonance imaging (MRI) sequence [24]. HT is a severe complication after thrombolysis which dramatically worsens patient outcome. Exploring strategies that can prevent HT occurrence and reduce hemorrhage volume is especially urgent for thrombolytic therapy. Given that HMGB1 has been reported to deteriorate neurovascular complications in stroke and might be elevated after tPA treatment evidenced in our present study, preventing HMGB1 signaling might provide a good strategy to attenuate the side effects caused by tPA. Here, we firstly demonstrate that HMGB1 blocking peptide reduced the tPA-associated mortality, brain hemorrhage, swelling, and BBB disruption in an animal model of ischemic stroke.

Extracellular HMGB1 in its reduced form with a disulfide bond connecting C23 and C45 exhibits a cytokine-inducing activity [25, 26] and participates in the pathological processes of numerous inflammatory and autoimmune diseases [25, 27]. Recently, it was paid particular attention on its significant role in brain ischemia/reperfusion. As one of the damage-associated molecular pattern molecules, extracellular HMGB1 binds to pattern recognition receptors of microglia and subsequently leads to synthesis and release of pro-inflammatory mediators, aggravating neuronal injury and BBB disruption [9]. In addition, extracellular HMGB1 also acts on its target receptors on endothelial progenitor cells (EPCs) to promote peri-infarct angiogenesis [28] which is related to ischemia/reperfusion-induced HT [29]. Here, we step forward to show that serum HMGB1 was increased in both patients and rats after tPA treatment and that blocking HMGB1 signaling significantly reduced neurovascular complications, emphasizing the critical role of HMGB1 in tPA-induced HT and the pathological importance during cerebral ischemia/reperfusion.

Brain ischemia/reperfusion can induce HMGB1 translocation and release. According to a previous report, HMGB1 level in cerebrospinal fluid was approximately twofold at 2 h, and increased to sixfold higher at 12 h after reperfusion in MCAO rats than sham-operated animals [14], indicating the time-dependent elevation of serum HMGB1 after reperfusion. To investigate tPA, rather than reperfusion time, induced serum HMGB1 increase, we collected patients’ sera at 2 h post-tPA treatment in order to reduce the effect caused by reperfusion time. Meanwhile, we compared the changes of HMGB1 in tPA and saline-treated MCAO rats within the same time durations. Our data revealed that serum HMGB1 levels seemed to be elevated after tPA treatment within a short time. In the MCAO rat model, serum HMGB1 level was also increased after tPA treatment, but did not obviously alter in vehicle-treated rats. It would be better to detect HMGB1 in cerebrospinal fluid where HMGB1 play the role in central nervous system diseases. Due to the technical limitation in our lab, serum HMGB1, rather than CSF HMGB1, was determined. How tPA treatment induces HMGB1 release is unknown. Some indirect evidences may imply this finding. For example, tPA could upregulate the expression of inflammatory mediators in cultured human cerebral microvascular endothelial cells [30], which might then mediate inflammatory cell death and the subsequent passive HMGB1 release.

Currently, there are several approaches to block HMGB1-mediated inflammation and thereby protecting neurovascular unit, including neutralizing monoclonal antibody (mAb) [13, 14], N-terminal domain of HMGB1 (competitively binds to receptors) [31], non-specific inhibitors like glycyrrhizin [32], and blocking peptide (HBHP) [15, 19, 33]. Among them, mAb requires intracerebroventricular injection, whereas HMGB1 N-terminal domain and glycyrrhizin lack target specificity. Fortunately, Kim et al. reported that HBHP binds directly to HMGB1 A box, confers anti-inflammation effect, and suppresses the synergistic effect of LPS and HMGB1 [33]. More importantly, intranasal delivery of low-dose HBHP effectively reduces infarct volume and improves neurological outcome after cerebral ischemia [15]. These elegant works suggest that HBHP would be a good drug candidate for stroke therapy. In the present study, intravenous injection of TAT-fused HBHP could pass BBB to get to brain parenchyma [34], maybe directly block HMGB1-mediated glial cell activation and inflammatory response, and subsequently dramatically reduce BBB disruption, HT and mortality after thrombolysis, although the drug bioactivity and pharmacological mechanisms are not fully observed and require in-depth investigation.

Since most 4.5 h-tPA rats who might have more amount of brain bleeding could not survive to 24 h, the results of hemoglobin quantification and BBB permeability detected in the survived rats at 24 h were inaccurate, and the results of surviving animals (24 h after MCAO) would have bias. Therefore, we calculated the hemoglobin contents of hemispheres again before the death time of most rats (8 h after ischemia), and found that tPA administration caused more severe hemorrhage, and further indicated the protective effect of HBHP on hemorrhagic transformation.

The loss of BBB leakage is a critical factor inducing HT after the ischemia/reperfusion [35]. Pretreatment determination of BBB permeability can predict post-treatment intracranial hemorrhage in patients receiving intravenous tPA or endovascular therapy [36, 37]. Meanwhile, HMGB1 was reported to exaggerate BBB insult [13, 14]. In line with these findings, we showed here, tPA exacerbated the extravasation of Evans blue and the impairment of tight junction proteins, the markers of BBB leakage. HBHP significantly attenuated tPA-mediated exaggeration of BBB impairment. However, an opposite finding was observed in an in vitro study, in which, HMGB-1 reduced tPA-driven BBB leakage [38]. The inconsistency might be explained by a much lower metabolic rate and the lack of micro-environment producing inflammatory factors in cell culture system compared with in vivo conditions.

Moreover, neuroinflammation is one of the main causes of BBB breakdown and HT [39]. Consistently, HBHP significantly alleviated the activated microglia and reduced the mRNA or protein levels of IL-1β, IL-6, IL-12b, iNOS, and COX-2 in the ischemic brains with tPA treatment. Considering the induction of iNOS, COX-2 has been reported to be involved in the disruption of BBB [40], our findings suggest that the activation of microglia and cytokine expression contribute to HMGB1-induced BBB disruption and HT development after thrombolysis in ischemic stroke. Therefore, it is reasonable that HBHP, which has the ability to reduce the expression of proinflammatory factors, exerted profound therapeutic effects on tPA-associated complications. In addition, the present study indicated that HBHP and purified HMGB1 could not directly interact with tPA (Additional file 2: Figure S2), possibly represents that they did not alter the protease activity. However, we could not exclude other potential underlying mechanisms that mediate this therapeutic effect of HBHP, such as the disturbance of other protein-tPA interaction. Clear mechanisms are expected to be uncovered in our following studies.

## Conclusions

In conclusion, the levels of serum HMGB1 seemed increased after thrombolysis in stroke patients and animals. Furthermore, HBHP treatment could reduce cerebral hemorrhage, brain swelling and mortality in tPA treatment after stroke. Our findings indicate that HMGB1-mediated inflammation and BBB breakdown may contribute to tPA-induced occurrence of HT and death in ischemic stroke, and that blocking HMGB1 signaling would be helpful to prevent the complications brought by thrombolysis. However, rationally designed clinical studies with well-defined patient population are needed to validate whether HBHP can be developed as an efficacious adjuvant therapy for ischemic stroke.

## Additional files

Additional file 1: **Figure S1.** Western blot analysis indicated serum HMGB1 levels were elevated after thrombolysis in stroke patients and rats. Sera from human and rats received thrombolysis were incubated with Protein A/G MagBeads (GenScript, Piscataway, NJ) to remove immunoglobins and then the proteins were separated in SDS-PAGE gels and immunolabeled with HMGB1 antibody. (PDF 340 kb)

Additional file 2: **Figure S2.** HBHP and HMGBP could not directly interact with tPA. Biolayer interferometry binding measurements using BLItz system from ForteBio were used to detect the binding affinity of HBHP and HMGBP with tPA. (A) Proteins were biotinylated and immobilized on streptavidin-coated biosensor tips. After equilibration, the tips were probed with the interacting analytes. The complexes were dissociated by immersing the sensor into sample dilution buffer. Data were generated automatically by the Octet User Software. (B) tPA binding measurements with HMGB1 were analyzed. The results showed that the combined signals were the same as those of uncured sensor, indicating HMGB1 has no specific binding with tPA. (C) HBHP binding measurements with tPA were analyzed. The combined signals were the same as those of the uncured sensor, indicating HBHP also has no specific binding with tPA. (PDF 331 kb)

## Abbreviations

BBB: Blood–brain barrier
ELISA: Enzyme-linked immunosorbent assay
EPCs: Endothelial progenitor cells
HBHP: HMGB1-binding heptamer peptide
HMGB1: High-mobility group box 1
HT: Hemorrhagic transformation
mAb: Neutralizing monoclonal antibody
MCAO: Middle cerebral artery occlusion
mNSS: Modified Neurological Severity Score
SLE: Systemic lupus erythematosus
tPA: Tissue-type plasminogen activator
TTC: 2,3,5-Triphenyltetrazolium chloride
