# Supplementary figures and images for: Cell permeable HMGB1-binding heptamer peptide ameliorates neurovascular complications associated with thrombolytic therapy in rats with transient ischemic stroke
Source: J Neuroinflammation. 2018 Aug 23;15:237. doi: 10.1186/s12974-018-1267-5 (PMC6108117; doi:10.1186/s12974-018-1267-5)

**human**

**HMGB1**

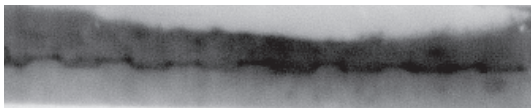

**25 kDa**

**pre-tPA**

**post-tPA**

**rats**

**HMGB1**

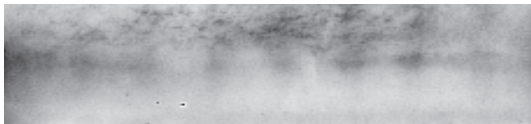

**25 kDa**

**pre-tPA**

**post-tPA**

Supplement: Supplementary file 1 — Figure S1. Western blot analysis indicated serum HMGB1 levels were elevated after thrombolysis in stroke patients and rats. Sera from human and rats received thrombolysis were incubated with Protein A/G MagBeads (GenScript, Piscataway, NJ) to remove immunoglobins and then the proteins were separated in SDS-PAGE gels and immunolabeled with HMGB1 antibody. (PDF 340 kb) [file 12974_2018_1267_MOESM1_ESM.pdf]
