# Supplementary material for: Cell permeable HMGB1-binding heptamer peptide ameliorates neurovascular complications associated with thrombolytic therapy in rats with transient ischemic stroke
Source: J Neuroinflammation. 2018 Aug 23;15:237. doi: 10.1186/s12974-018-1267-5 (PMC6108117; doi:10.1186/s12974-018-1267-5)
Supplement: Supplementary file 2 — Figure S2. HBHP and HMGBP could not directly interact with tPA. Biolayer interferometry binding measurements using BLItz system from ForteBio were used to detect the binding affinity of HBHP and HMGBP with tPA. (A) Proteins were biotinylated and immobilized on streptavidin-coated biosensor tips. After equilibration, the tips were probed with the interacting analytes. The complexes were dissociated by immersing the sensor into sample dilution buffer. Data were generated automatically by the Octet User Software. (B) tPA binding measurements with HMGB1 were analyzed. The results showed that the combined signals were the same as those of uncured sensor, indicating HMGB1 has no specific binding with tPA. (C) HBHP binding measurements with tPA were analyzed. The combined signals were the same as those of the uncured sensor, indicating HBHP also has no specific binding with tPA. (PDF 331 kb) [file 12974_2018_1267_MOESM2_ESM.pdf]

A

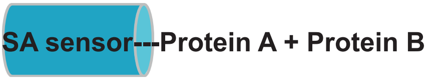

B

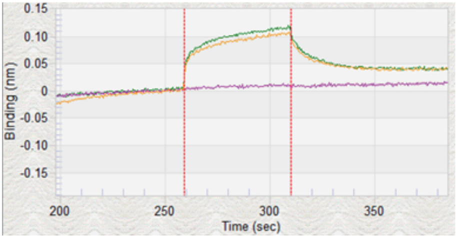

Imobilize tPA and analyze HMGB1
Mobilize tPA and analyze HMGB1

C

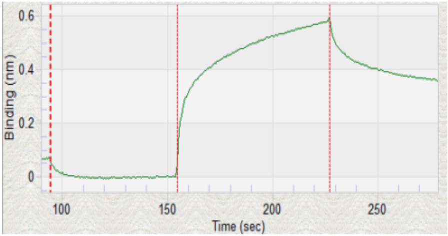

Mobilize HBHP and analyze tPA

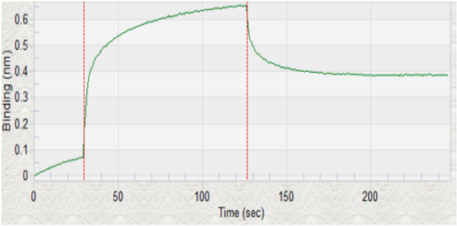

Imobilize HBHP and analyze tPA
